# ANGIOLYMPHOID HYPERPLASIA WITH EOSINOPHILIA: ATYPICAL APPEAREANCE IN AN OLDER PATIENT

**DOI:** 10.4103/0019-5154.43206

**Published:** 2008

**Authors:** Ozlem Karabudak, Oktay Taskapan, Onder Bozdogan, Bilal Dogan

**Affiliations:** *From the Department of Dermatology, GATA Teaching Hospital, Tibbiye Street 81327 Kadikoy, Istanbul, Turkey*; 1 *From the Department of Pathology, Kirikkale University Faculty of Medicine, Istanbul, Turkey*

**Keywords:** *Angiolymphoid hyperplasia with eosinophilia*, *non-healing ulcer*

## Abstract

We describe a 76-year-old man presenting with a chronic, non-healing ulcer of six-year duration on his left zygomatic area. The skin biopsy specimen taken from the lesion, showed increased vascular proliferation, edematous endothelial cells in the dermal blood vessels and perivascular eosinophilic/lymphocytic infiltration. The routine and specific blood tests were unremarkable. On the basis of these features, the patient was diagnosed as having angiolymphoid hyperplasia with eosinophilia (ALHE). We present the case because of its rarity in older people, atypical clinical appearance; and stress the consideration of ALHE in the differential diagnosis of chronic non-healing superficial ulcers confined to face and neck.

## Introduction

Angiolymphoid hyperplasia with eosinophilia (ALHE) is an uncommon idiopathic condition characterized isolated or grouped papules, plaques, or nodules in the skin of the head and neck. ALHE is a benign neoplasm, but may be persistent and difficult to eradicate. It presents most commonly in patients aged 20-50 years and rarely in elderly people.

## Case History

**Fig. 1:**
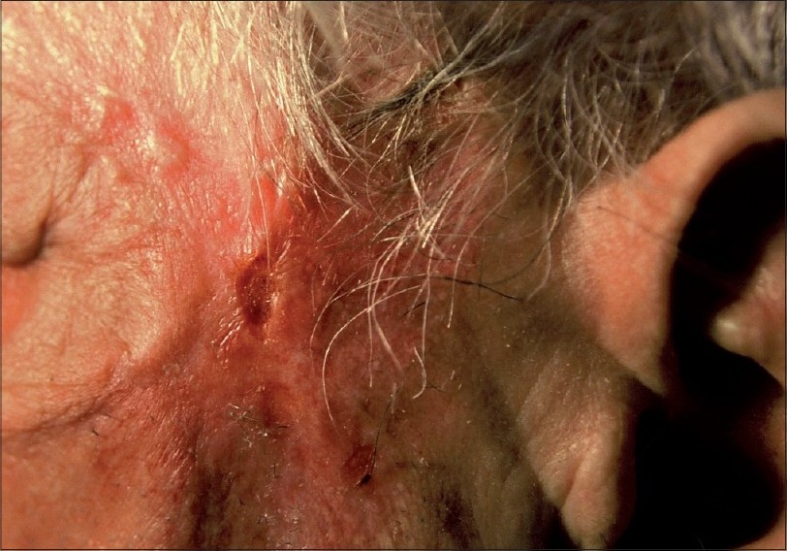
Ulcerated plaque in the left zygomatic area

A 76-year-old man presented with a chronic, non-healing ulcer of six-year duration on his left zygomatic area. His medical history was unremarkable except hypertension controlled with anti-hypertensive medication (amlodipine, 5mg/day). No abnormality was detected in general physical examination. Dermatological examination revealed an asymptomatic, atrophic, partly crusted and ulcerated plaque of 8 × 3 cm in diameter in the left zygomatic area (Fig. 1).

**Fig. 2:**
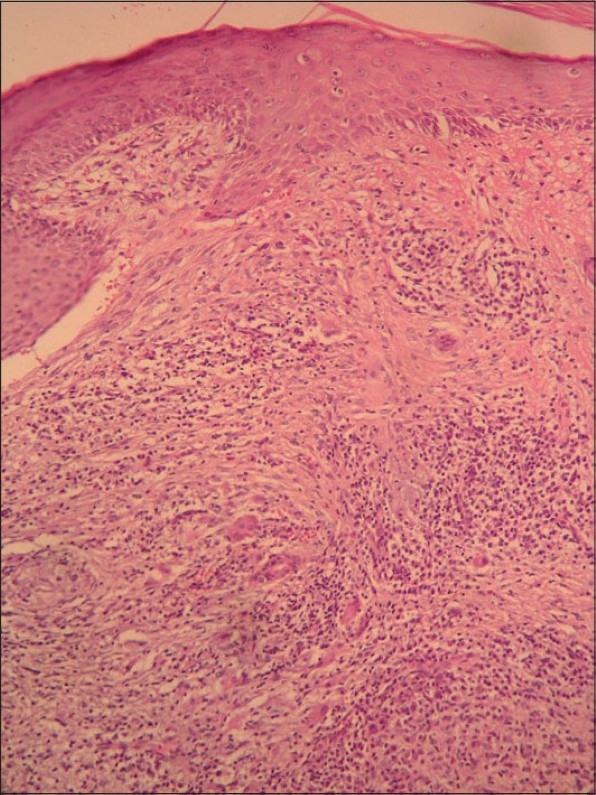
Dense chronic inflammation and vascular network in dermis. (H&E, ×20)

**Fig. 3:**
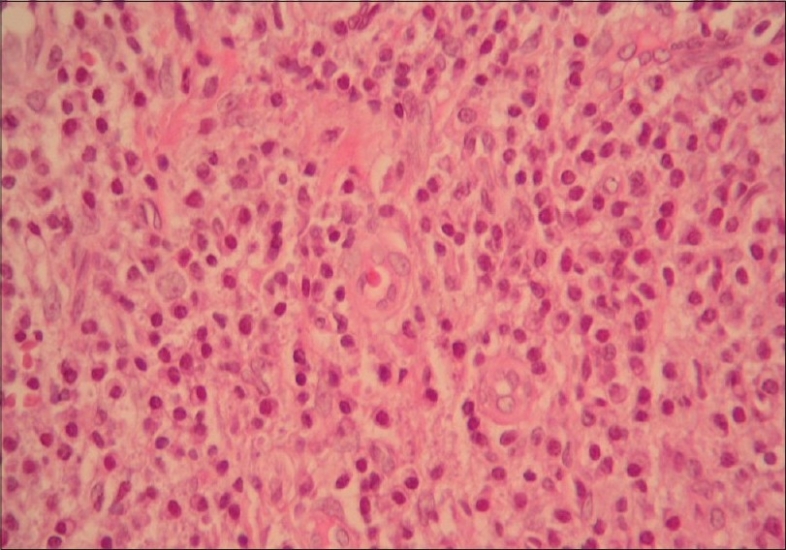
Prominent histiocyte-like endothelial cells and chronic inflammatory cells (eosinophils and neutrophils) around vessels. (H&E, ×40)

A biopsy specimen was obtained from the lesion (Figs. 2 and 3).

Histopathologic examination of the skin biopsy specimen showed increased vascular proliferation, edematous endothelial cells in the dermal blood vessels and perivascular eosinophilic/lymphocytic infiltration. All routine laboratory investigations including whole blood count, sedimentation rate, renal and liver function tests, peripheral blood eosinophil count and serum total Ig E level were within normal limits. On the basis of clinical and histopathological features, the patient was diagnosed as having ALHE. He was treated with cryotherapy. However, there was a recurrence in a few months. The patient didn't come to regular follow-up examinations.

## Discussion

Angiolymphoid hyperplasia with eosinophilia, first described by Wells and Whimster in 1969, is a rare benign vascular tumor.^1^ It is characterized by one or more purplish, brownish papules and subcutaneous nodules with a predilection for the head and neck regions. Other tissues such as orbit, heart, bone, liver and spleen may also be involved. It is more common in middle-aged females. Contrary the name suggests, peripheral blood eosinophilia is not a constant finding in ALHE.^2^ Histopathologically, ALHE is characterized by numerous thick and thin-walled vessels lined with characteristic edematous endothelial cells (“hobnail” or “tombstone” appearance) associated with variable lymphocytic and eosinophilic infiltrate.^3^

The etiopathogenesis of ALHE is not well known. Trauma, hormonal changes and infections (HTLV or HHV 8) have been suggested to play a role in the pathogenesis.^4^ Association of ALHE with nephrotic syndrome and pregnancy have been described.^4^,^5^ Over-expression of estrogen and progesterone receptors was detected in pregnant women.^6^ Interleukin 5 and vascular endothelial growth factor were also found to be increased in some cases.^7^ Since they share many clinical and histopathological similarities, ALHE should be differentiated from Kimura's disease. Kimura's disease occurs in younger patients, the lesions show deeper localization and association with lymphadenopathy. Histologically, it contains sclerosis at any stage, but does not have epithelioid endothelial cells.^8^

The clinical appearance of the lesion in our patient resembles sclerosing (morpheaform) basal cell carcinoma (BCC) which is characterized by a yellowish-whitish sclerotic plaque with poorly defined margins and induration.^9^ Our patient's lesion had no signs regarding sclerosis and induration. In addition, ALHE and sclerosing BCC show completely different histopathological patterns.

The most common therapeutic options are surgical excision and pulsed dye laser. Cryotherapy, irradiation, intralesional steroid injection, carbon dioxide laser have also been reported as therapeutic options with variable levels of success.^10^

The older patient of ALHE presented here had an atypical appearance. We suggest that ALHE should be considered in the differential diagnosis of chronic non-healing superficial ulcers confined to face and neck.
